# Progressive multifocal leukoencephalopathy in a patient with B-cell chronic lymphocytic leukemia after COVID-19 vaccination, complicated with COVID-19 and mucormycosis: a case report

**DOI:** 10.1186/s12883-024-03650-y

**Published:** 2024-05-04

**Authors:** Hamed Amirifard, Mojtaba Shahbazi, Ghasem Farahmand, Zahra Ranjbar, Maryam Kaeedi, Sanaz Heydari Havadaragh

**Affiliations:** 1 Iranian Center of Neurological Research, Neuroscience Institute, Tehran University of Medical Sciences, Tehran, Iran; 2 Neurology, Iranian Center of Neurological Research, Tehran University of medical sciences, Imam Khomeini Hospital Complex, Tehran, Iran

**Keywords:** B-CLL, PML, COVID-19, Mucormycosis

## Abstract

**Background:**

Progressive multifocal leukoencephalopathy (PML) is a rare and fatal opportunistic viral demyelinating infectious disease of the central nervous system (CNS). There are various clinical presenting symptoms for the disease.

**Case presentation:**

This paper presents a clinical case of PML in a patient with B-Chronic lymphocytic leukemia (B-CLL), previously treated with Chlorambucil, later complicated later with COVID-19 and mucormycosis.

**Conclusion:**

PML can develop in the setting of cellular immune dysfunction. Late diagnosis of this disease based on nonspecific symptoms is common, therefore when we face a neurological complication in a CLL or immunocompromised patient, we should consider PML infection. A remarkable feature of this case is the possible triggering effect of COVID-19 vaccination for emergence of PML as the disease can be asymptomatic or sub-clinical before diagnosis.

## Introduction

Progressive multifocal leukoencephalopathy (PML) is a rare and fatal opportunistic viral demyelinating infectious disease of the central nervous system (CNS), initially described in 1958 in patients with lymphoproliferative and myeloproliferative diseases [1, 2].

The John Cunningham polyomavirus (JCV) is known as the causative agent for development of PML. Reactivation of this virus almost always occurs in the context of a major cell-mediated immune system suppression. JCV is a ds-DNA ubiquitous human pathogen with both inhalation and ingestion transmission routes. Contamination with this virus is presumably asymptomatic; thereafter the second phase would be a variable long period of latent infection, most affecting the kidneys, bone marrow, spleen and B lymphocytes. The third or final stage is the reactivation and dissemination of the virus into the CNS in the setting of cellular immunity impairment. Of note, cellular immunity suppression is a potent risk factor for PML occurrence rather than humoral immunity impairment [3].

JC virus antibodies commonly have been found in about 86% of adults after initial asymptomatic exposure to the virus in childhood [4]. Reactivation of JCV occurs in immunocompromised individuals with human immunodeficiency virus (HIV)/ human T-lymphotropic virus type 1 (HTLV1), hematologic malignancies, organ transplantation, or treatment with immunosuppressive agents (e.g. monoclonal antibodies such as natalizumab). Malignancy is one of the most common predisposing conditions among PML cases [5].

Population-based epidemiologic studies of PML are scarce; most of them have reported the incidence in specific populations at risk. Since the beginning of the SARS-COV-2 pandemic, the relationship between this virus and PML in the literature is not clearly identified.

Here we are reporting a clinical case of PML in a patient with B cell chronic lymphocytic leukemia (B-CLL), previously treated with Chlorambucil, further complicated with COVID-19 and mucormycosis. Only a few cases of PML following Chlorambucil treatment have been described in the literature. Another challenging issue in our patient was his early PML manifestation following COVID-19 vaccination, complicating the diagnosis.

## Case presentation

The patient was a 68-year-old-man admitted to our tertiary referral hospital with complaints of progressive drowsiness and confusion from 2 months before.

In 2011, the patient was diagnosed with B-CLL through the detection of a long arm deletion of chromosome 13 at position 14 via fluorescence in situ hybridization (FISH), bone marrow aspiration and biopsy, flow cytometry, and immunohistochemistry (IHC). Initially, he didn’t receive treatment as being asymptomatic until 2015, when cyclical chlorambucil was started only until 2020. He also had a documented history of basal cell carcinoma diagnosed in a facial papule which was resected with a normal margin 2 years before without any local recurrence.

The patient was doing well until August 2021 when he presented with fever, imbalance, and restlessness 4 days after the second dose of the Sinopharm vaccine (second 0.5 mL intramuscular injection of the inactivated virus). Initially his fever subsided but he gradually started to experience memory disturbances, difficulty keeping focus and finding directions accompanied by behavioral and personality changes.

**Fig. 1:**
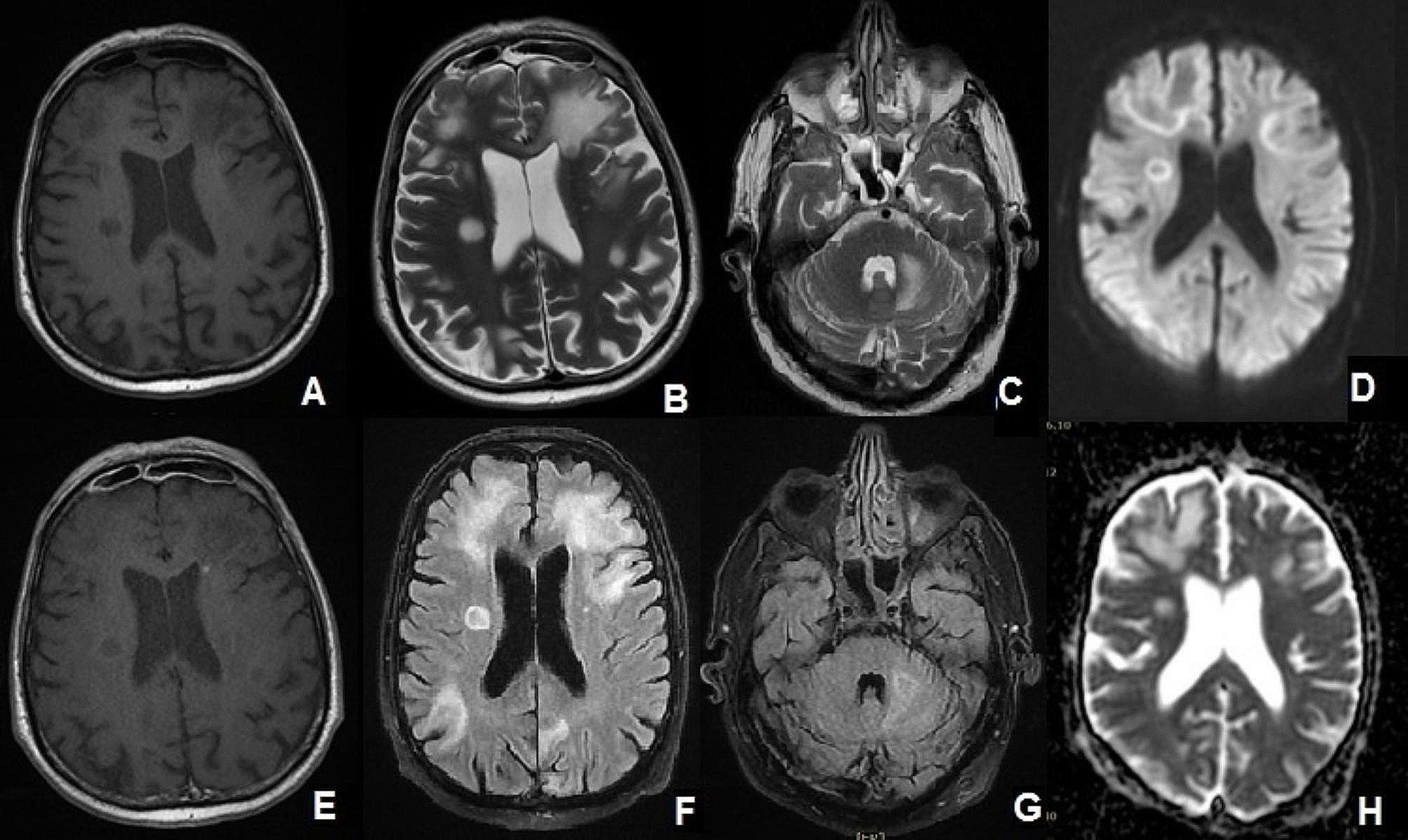
Brain MRI +/- GAD: **A**: in T1 sequence multiple hypointense and well-defined lesions in frontal, parietal, basal ganglia are seen. **B**, **C**, **F**, and **G**: T2/FLAIR sequences shows multiple hyperintense lesions in frontal, parietal, occipital, basal ganglia and cerebellum with sharp borders toward gray matter contrasting with ill-defined borders toward white matter. Subcortical lesions involve U-fibers. **D**, **H**: In DWI and ADC sequences peripheral restriction in noted. **E**: In post Gadolinium injection sequences; hazy peripheral punctate enhancement in frontal region is present. Evidence of mucosal thickening in bilateral paranasal sinuses in favor of mucormycosis sinusitis also seen (proved by pathological biopsy report)

**Fig. 2:**
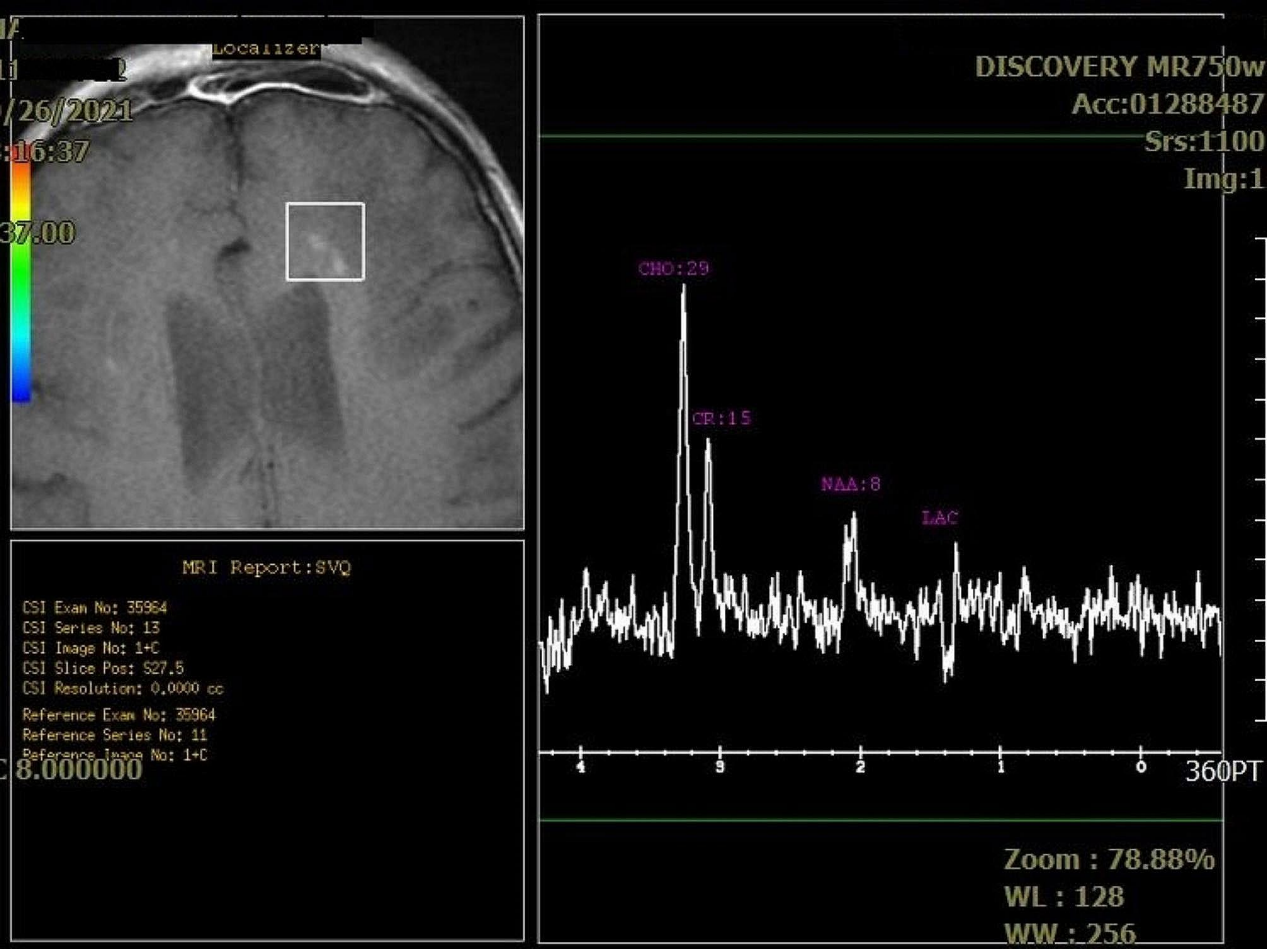
Brain MRS: Mild decrease in NAA and also in Choline in abnormal mentioned lesion was seen. Choline/Creatine Ratio was about 0.8, excluding the possibility of neoplastic lesions (primary or secondary) and suggesting of demyelinating/inflammatory process like PML

In late September, urinary and stool incontinence were added to the symptoms and he was admitted for a primary evaluation in another center; there empirical treatment and evaluation for meningoencephalitis was initiated. At the time of admission to our tertiary center, the patient was drowsy, answering questions with short sentences and was confused especially in performing multi-step tasks. In the Mini- Mental State Exam (MMSE) his score was 17 points (mostly impaired in recall and calculation parts). He wasn’t febrile and a general examination revealed no obvious meningismus or lymphadenopathy. In neurological examination, cranial nerves were intact, and no hemiparesis or hemi-sensory loss was detected while having an unsteady gait. Plantar reflexes were both downward. Brain computed tomography (CT) scan showed multiple areas of hypo-densities without any mass effect. On brain Magnetic resonance imaging (MRI), T2/FLAIR sequences revealed relatively large areas of intensity alteration and gliosis at subcortical white matter of both parieto-occipital lobes. Similar changes in the left hemisphere of the cerebellum as well as in the midbrain were seen (Fig. 1). In diffusion weighted image (DWI) sequences, the lesions showed peripheral restriction and in post gadolinium T1 sequence, hazy peripheral punctate enhancement in the frontal region was seen. Magnetic resonance spectroscopy (MRS) revealed a mild decrease in N-acetyl aspartate (NAA) and also in Choline in the abnormal area. Choline/Creatine Ratio was about 0.8, excluding the possibility of neoplastic lesions (primary or secondary) and suggesting of demyelinating/inflammatory process like PML (Fig. 2).

Laboratory tests showed an abnormal CBC in the form of anemia and thrombocytopenia (White blood cell: 36,000 (95% lymphocytes), (Hemoglobin: 10.9 g/dl; Mean corpuscular volume: 88.5) (platelet count: 91,000)). Liver, renal and thyroid function tests, serum electrolytes and C-reactive protein (CRP) all reported within normal limits. The encephalitis panel in Cerebrospinal fluid (CSF) for viral, bacterial and fungal etiologies (HIV, HSV1, HSV2, CMV, TB, Cryptococcus, Toxoplasmosis), blood vasculitis markers (antinuclear antibody, anti-double stranded DNA (Anti-dsDNA) antibodies, antiphospholipid antibody, anticardiolipin antibody, rheumatoid factor) and also viral markers (hepatitis B surface antigen (HBsAg), Hepatitis C Virus (HCV) antibody and human immunodeficiency virus (HIV) antibody tests) reported negative. Also, at the beginning of his admission COVID-19 PCR was negative. Serologic evaluation for Brucella and toxoplasmosis IgM were negative.

A second lumbar puncture showed normal cell count, protein (0.28 mg/dL) and glucose. There were no abnormal cells on direct smear and fungal evaluation. No abnormal cell was detected in the CSF cytology and CSF cytometry was negative. JCV PCR was positive in the CSF. Consequently, the diagnosis of PML was made.

**Fig. 3:**
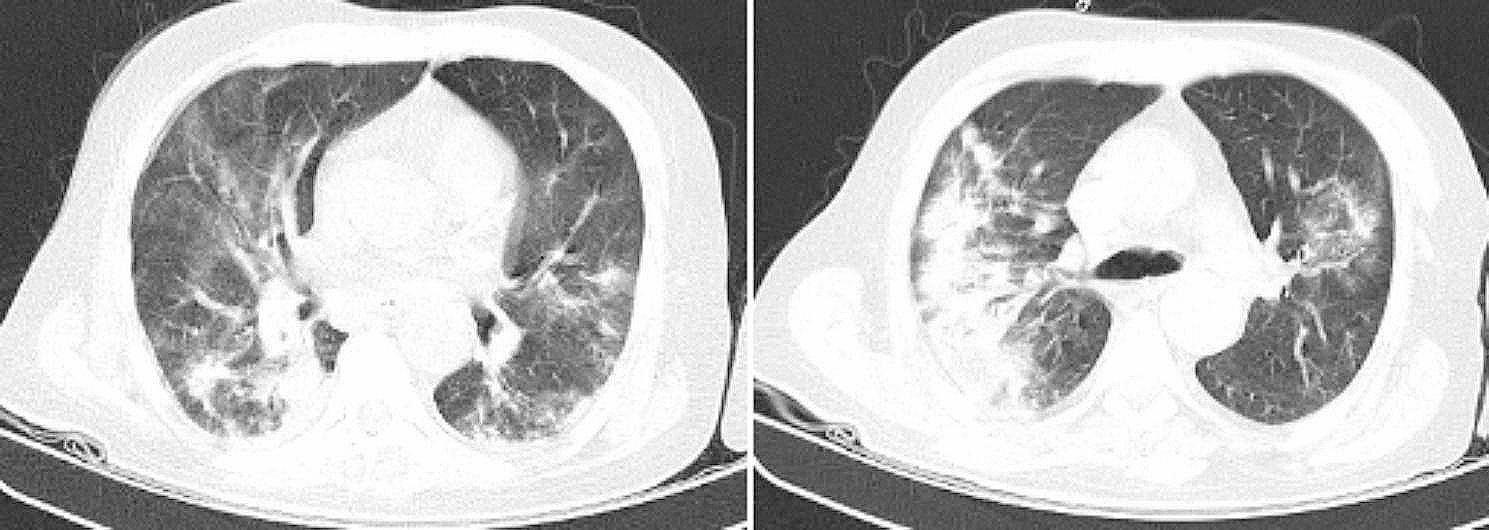
Chest CT scan shows multiple patchy ground glass opacities in favor of Covid-19 (Confirmed by PCR)

Unfortunately, during hospitalization, the patient’s level of consciousness gradually worsened leading to concerns of COVID-19 involvement considering the existing pandemic. This concern was later, proven with a second chest CT scan and a positive COVID-19 PCR (Fig. 3).

**Fig. 4:**
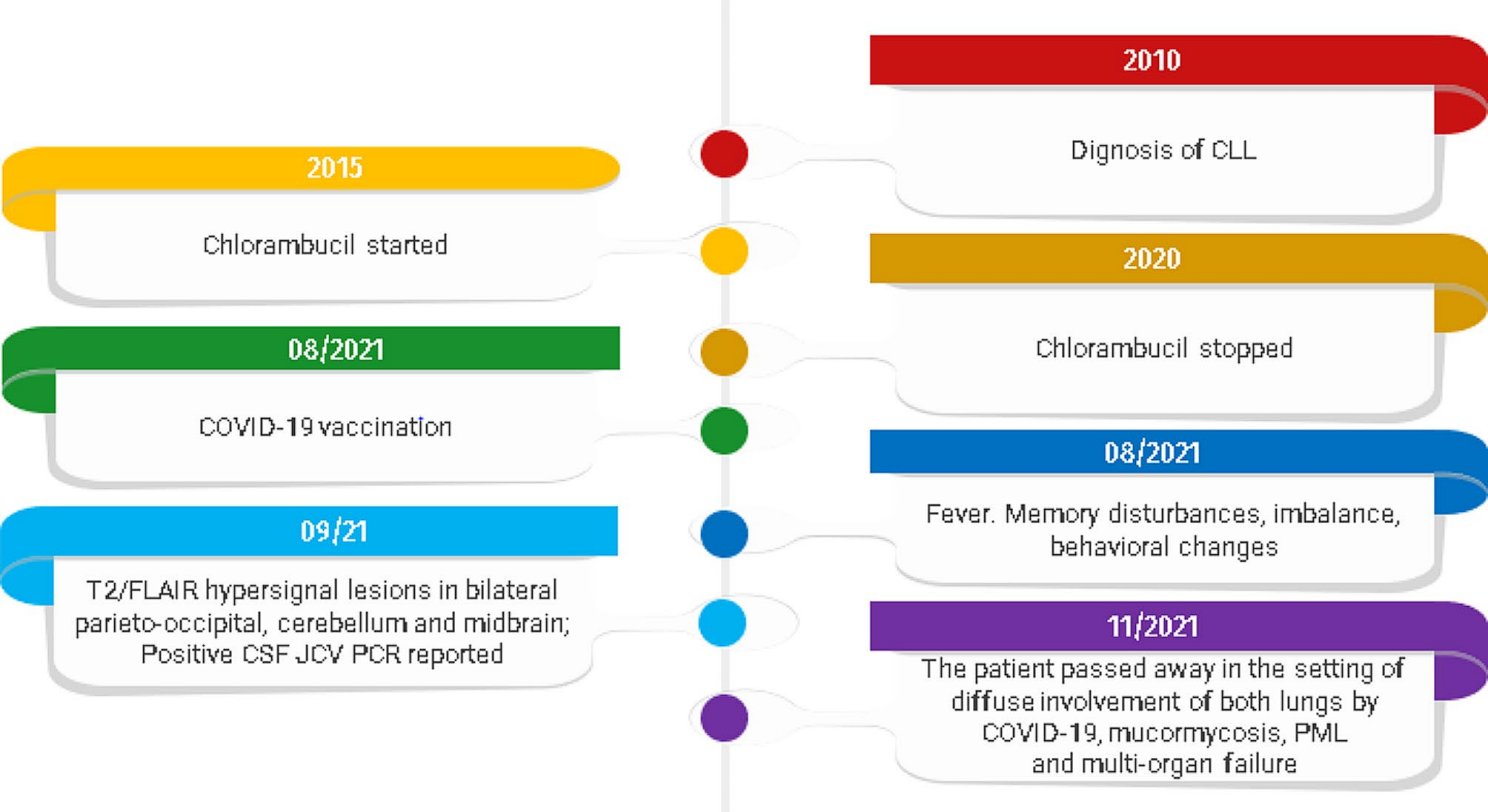
The timeline of the patient’s disease

While under conservative and supportive treatment for COVID-19 infection, he revealed early signs of rhinosinusitis. Ear, nose, and throat (ENT) evaluation with nasal direct smear demonstrated septate hyphae, suggesting mucormycosis involvement as the patient was predisposed due to underlying immunosuppression, diabetes mellitus with recently poor blood glucose control. Despite all efforts for treatment, the patient eventually passed away of multi-organ failure after 3 months of his initial symptoms (Fig. 4).

## Discussion

Multiple overlapping risk factors and underlying conditions are involved in PML occurrence, including background diseases (e.g. hematologic malignancies, immunosuppressive agents, etc.) [6]. Hematologic malignancies, such as Hodgkin and Non-Hodgkin lymphoma (NHL), Waldenstrom macroglobulinemia, myeloma and Chronic lymphocytic leukemia (CLL) are the most commonly seen malignancies associated with PML, as well as in conditions such as hematopoietic stem cell transplantation (HSCT) [7]. In previous cohorts, the incidence rate of PML in NHL and CLL had been reported 8.3 (95% CI 1.71–24.24) and 11.1 (0.28–61.74) per 100,000 person-years respectively [8]. Only few cases of Chlorambucil-induced PML in CLL are described in literature [9, 10].

There are various presenting clinical symptoms for the disease: usually, it begins with focal or multifocal neurological deficits that progress in a sub-acute course leading to serious disabilities or even death. Sometimes the acute onset of symptoms may mimic other neurological diagnoses like stroke [11]. There are few case reports of completely asymptomatic courses of the disease in the literature [12]. According to evidence, delayed diagnosis in PML cases is not uncommon [13].

PML was, initially introduced as a white matter progressive demyelinating multifocal disease due to its pathological characteristics. Over time, diagnostic criteria have evolved. With the detection of JCV DNA in CSF along with congruent clinical symptoms and radiological features, making the diagnosis of PML is possible without a biopsy [14]. In brain MRI of these patients, multifocal and mostly asymmetric lesions are seen in periventricular and subcortical regions with some of them may having minimal mass effect or enhancement. Previous studies have shown more common cerebral rather than brain stem involvement with a ratio of 10:1 [15].

Early detection of PML especially in the asymptomatic phase is associated with a more favorable outcome and survival rate; although the disease is devastating condition nevertheless with a significant risk of long-term mortality and morbidity [16]. There is no specific therapy for PML at the time of reporting this case, and the treatment mainly consists of supportive care. Building up the immune system is the mainstay of treatment for PML [17].

As mentioned, the main predisposing factor in our case was immunosuppression in the background of CLL disease. Impairment of CD4 differentiation into Th1 cells and decreased CD8 T cell cytotoxicity are the result of significant alterations of T lymphocyte gene expression seen in CLL patients [7]. Immune system dysfunction may also lead to several opportunistic viral and fungal infections. It is possible that, the simultaneous infection of PML and COVID-19 is a result of T cell dysfunction in the presented patient.

Based on the literature, anti-JCV seropositivity of virtually all patients with PML related to Natalizumab therapy and the positive correlation of antibody titers with disease incidence rates, altogether demonstrate the ineffectiveness of the humoral immune response to prevent reactivation of JCV [5]. Thus, T cells are seemingly the principal immune cells in defense against JCV, like other viruses in an immune-competent condition. This issue is also recently suggested in COVID-19 patients, in which a dissociation between severity and seroconversion has been reported [18].

Mucormycosis (black fungus) is a rare life-threatening fungal infection occurring in immunocompromised individuals. After the COVID-19 pandemic (on March 2020), due to the frequent use of corticosteroids, as well as the presence of uncontrolled hyperglycemia that often accompany the viral infection, the risk of this superimposed infection increased [19–21]. In addition, in CLL disease, despite an increased number of WBCs, functional impairment of these cells leads to substantial immunosuppression that may result in PML and Mucormycosis infections.

In our patient, considering the underlying low-grade CLL previously treated only with Chlorambucil and presentation of symptoms shortly after COVID-19 vaccination, the diagnostic challenge was to exclude infiltration of CLL which could mimic PML alongside post-vaccination inflammation phenomena like Acute disseminated encephalomyelitis (ADEM). Our presented case of PML was complicated with COVID and mucormycosis infection leading to inevitable death. Another notable feature is that COVID-19 vaccination may act as a trigger for emergence of PML in this patient as the disease can remain asymptomatic or subclinical before eventual diagnosis. This feature resulted in the development of screening protocols for the detection of virus and its antibody titers in multiple sclerosis (MS) patients receiving Natalizumab.

## Conclusion

As seen in this patient, PML can develop in the setting of cellular immune dysfunction. Delayed diagnosis of this disease based on nonspecific symptoms is common, therefore when facing neurological complication in an immunocompromised patient, we should consider PML infection. It is unclear if there is any association between COVID-19 vaccine and PML. To the best of our knowledge, no case of post COVID-19 vaccination PML has been reported to this date. We hypothesize that the temporal association of PML’s clinical manifestation after receiving the COVID-19 vaccine may suggest a triggering role in PML reactivation.

## Data Availability

The datasets generated and/or analyzed during the current study are not publicly available due the nature of the study but are available from the corresponding author on reasonable request.
